# Mixed Communities of Mucoid and Nonmucoid *Pseudomonas aeruginosa* Exhibit Enhanced Resistance to Host Antimicrobials

**DOI:** 10.1128/mBio.00275-18

**Published:** 2018-03-27

**Authors:** Sankalp Malhotra, Dominique H. Limoli, Anthony E. English, Matthew R. Parsek, Daniel J. Wozniak

**Affiliations:** a Department of Microbial Infection and Immunity, The Ohio State University, Columbus, Ohio, USA; b Department of Microbiology and Immunology, Geisel School of Medicine, Dartmouth, Hanover, New Hampshire, USA; c Department of Microbiology, University of Washington, Seattle, Washington, USA; d Department of Microbiology, The Ohio State University, Columbus, Ohio, USA; University of Pittsburgh School of Medicine

**Keywords:** *Pseudomonas aeruginosa*, alginate, antimicrobial peptides, catalase, exopolysaccharide, polymicrobial

## Abstract

*Pseudomonas aeruginosa* causes chronic pulmonary infections in patients with cystic fibrosis (CF). *P. aeruginosa* mucoid conversion, defined by overproduction of the exopolysaccharide alginate, correlates with accelerated decline in CF patient lung function. Recalcitrance of the mucoid phenotype to clearance by antibiotics and the immune response is well documented. However, despite advantages conferred by mucoidy, mucoid variants often revert to a nonmucoid phenotype both *in vitro* and *in vivo*. Mixed populations of mucoid isolates and nonmucoid revertants are recovered from CF lungs, suggesting a selective benefit for coexistence of these variants. In this study, cocultures of mucoid and nonmucoid variants exhibited enhanced resistance to two host antimicrobials: LL-37, a cationic antimicrobial peptide, and hydrogen peroxide (H_2_O_2_). Alginate production by mucoid isolates protected nonmucoid variants in consortia from LL-37, as addition of alginate exogenously to nonmucoid variants abrogated LL-37 killing. Conversely, nonmucoid revertants shielded mucoid variants from H_2_O_2_ stress via catalase (KatA) production, which was transcriptionally repressed by AlgT and AlgR, central regulators of alginate biosynthesis. Furthermore, extracellular release of KatA by nonmucoid revertants was dependent on *lys*, encoding an endolysin implicated in autolysis and extracellular DNA (eDNA) release. Overall, these data provide a rationale to study interactions of *P. aeruginosa* mucoid and nonmucoid variants as contributors to evasion of innate immunity and persistence within the CF lung.

## IMPORTANCE

*P. aeruginosa* mucoid conversion within lungs of cystic fibrosis (CF) patients is a hallmark of chronic infection and predictive of poor prognosis. The selective benefit of mixed populations of mucoid and nonmucoid variants, often isolated from chronically infected CF patients, has not been explored. Here, we show that mixed-variant communities of *P. aeruginosa* demonstrate advantages in evasion of innate antimicrobials via production of shared goods: alginate and catalase. These data argue for therapeutically targeting multiple constituents (both mucoid and nonmucoid variants) within diversified *P. aeruginosa* communities *in vivo*, as these variants can differentially shield one another from components of the host response.

## INTRODUCTION

Cystic fibrosis (CF) is one of the most common lethal genetic diseases (1, 2). CF patients exhibit impaired mucociliary clearance, leading to recurrent pulmonary infections (3). During later stages of disease, the Gram-negative bacterium *Pseudomonas aeruginosa* predominates in the CF lung, exacerbating pathology and hastening patient mortality (4). *P. aeruginosa* infection promotes excessive influx of neutrophils into the lung, driving tissue damage, fibrosis, and organ dysfunction (5, 6). CF neutrophils overproduce antimicrobials such as reactive oxygen species (ROS) (e.g., hydrogen peroxide [H_2_O_2_] and hypochlorite [HOCl]) that damage host tissues and bacteria (7). Neutrophils and CF lung epithelium also secrete cationic antimicrobial peptides (AMPs) in excess (8, 9). One such AMP, LL-37, is a multifunctional cathelicidin that is bactericidal and immunomodulatory (10).

Importantly, exposure to sublethal concentrations of ROS and AMPs promotes bacterial mutagenesis and mucoid conversion, a critical *P. aeruginosa* pathoadaptation (11–13). The mucoid phenotype in *P. aeruginosa* is defined by overproduction of the polyanionic exopolysaccharide alginate (14). Nonmucoid, environmental isolates of *P. aeruginosa* initially colonize CF patients (15). However, exposure to host-derived mutagens (e.g., H_2_O_2_ and LL-37) promotes mutation of *mucA* (see Fig. S1 in the supplemental material), which encodes a transmembrane anti-sigma factor that sequesters its cognate sigma factor, AlgT (also known as AlgU or σ^22^) (16, 17). *mucA* mutation liberates AlgT, which promotes enhanced transcription of the alginate biosynthetic operon [*algD*(PA3540)-*algA*(PA3551)], and genes encoding ancillary transcription factors essential for alginate biosynthesis: *algB*, *algR*, and *amrZ* (18–20). Though AlgT is critical for mucoid conversion, the AlgT regulon is predicted to consist of 293 open reading frames, indicating a broad role in *P. aeruginosa* gene regulation (21, 22).

10.1128/mBio.00275-18.2 FIG S1  Paradigm for *P. aeruginosa* mucoid conversion and reversion to nonmucoid phenotype during chronic CF infection. The CF lung is first colonized with nonmucoid *P. aeruginosa* progenitors (*mucA*^*+*^ *algT*^*+*^). Via exposure to host-derived mutagens *in vivo* (e.g., neutrophils and neutrophil effectors, H_2_O_2_ and LL-37) *P. aeruginosa* acquires a mutation in *mucA* and converts to a mucoid phenotype (*mucA* mutant [“*mucA-*”] *algT*^*+*^), which is defined by overproduction of the exopolysaccharide alginate. Nonmucoid revertants (*mucA* mutant and *algT* mutant [“*algT-*”]) of *P. aeruginosa* can arise spontaneously from a subset of mucoid variants via second-site, suppressor mutations in *algT*, which encodes the sigma factor essential for upregulation of the alginate biosynthetic operon. Both mucoid variants and nonmucoid revertants are often coisolated from CF patients chronically infected with *P. aeruginosa*. Download FIG S1, TIF file, 0.2 MB. Copyright © 2018 Malhotra et al. 2018 Malhotra et al. This content is distributed under the terms of the Creative Commons Attribution 4.0 International license.

Mucoid conversion is correlated with decline in CF patient lung function and marks a transition to the progressively debilitating stages of disease (23). Compared to nonmucoid isolates, mucoid *P. aeruginosa* exhibits enhanced resistance to multiple antibiotics (24–27) and to host immune effectors (12, 28, 29). Apart from the alginate polysaccharide, *P. aeruginosa* expresses multiple factors that enable evasion of the host response, including proteases, rhamnolipids, and lipases (30). Additionally, *P. aeruginosa* catalases, encoded by *katA* and *katB*, are critical virulence factors that neutralize H_2_O_2_ stress (31, 32).

Despite recalcitrance of mucoid *P. aeruginosa*, both mucoid and nonmucoid variants are often isolated together from CF lung specimens (33–37). The majority of nonmucoid *P. aeruginosa* variants present within the CF lung in late disease have reverted from mucoid strains (Fig. S1) (33). These revertants predominantly arise via spontaneous suppressor mutations in *algT in vitro* (*mucA* and *algT* mutants) (38, 39). The propensity of mucoid *P. aeruginosa* to revert to the nonmucoid phenotype has been attributed to energetic costs of alginate production, which may be disadvantageous under certain conditions (39–41).

However, host factors selecting for nonmucoid revertants *in vivo* are not known. In light of benefits conferred by mucoidy, the copresence of nonmucoid revertants within hyperinflammatory CF airways suggests both variants contribute to *P. aeruginosa* persistence. As such, in this study, we hypothesized that mixed populations of mucoid and nonmucoid variants have an advantage in evading innate antimicrobials, wherein both *P. aeruginosa* morphotypes exhibit differential mechanisms to combat host factors. Indeed, we show when grown in consortia, mucoid and nonmucoid variants demonstrate enhanced resistance to LL-37 and H_2_O_2_. Each *P. aeruginosa* phenotype contributes a portion of immune protection, benefiting the community as a whole: mucoid variants protect both themselves and nonmucoid variants from LL-37 stress via alginate production. Conversely, nonmucoid revertants protect themselves and mucoid variants from H_2_O_2_ via catalase (KatA) production. We demonstrate *katA* is transcriptionally repressed when AlgT is active, via downstream transcription factor, AlgR. Additionally, extracellular release of catalase depends on *lys*, which mediates autolysis and extracellular DNA (eDNA) release. In total, these data provide important insights regarding mixed-variant *P. aeruginosa* interactions that enable evasion of critical components of host immunity.

## RESULTS

### Cocultures of mucoid and nonmucoid isolates exhibit enhanced resistance to host antimicrobials.

To determine whether there is a selective advantage of mixed mucoid and nonmucoid *P. aeruginosa* populations in evading host effectors, we focused on two innate antimicrobials found within the CF lung: LL-37 and H_2_O_2_ (7–9). We exposed monocultures or cocultures of mucoid and nonmucoid variants to either LL-37 or H_2_O_2_ for 1 h, followed by plating for colony-forming units (CFU). The clinical mucoid isolate FRD1 (*mucA* mutant) and isogenic nonmucoid strains (FRD1 *algD* or FRD1 *algT*) were differentially drug marked by streptomycin and rifampin (RIF), respectively, to independently track their survival in coculture. *algD*, the first gene in the alginate biosynthetic operon, encodes a GDP-mannose dehydrogenase essential for alginate production (18); FRD1 *algD* is an *algD* insertional mutant. FRD1 *algT*, which harbors both a *mucA* mutation and point mutation in a sigma-factor-encoding gene, *algT*, was first isolated as a spontaneous nonmucoid revertant of FRD1 (42) (see Table S1 in the supplemental material).

10.1128/mBio.00275-18.8 TABLE S1  All strains, plasmids, and primers used in this study. Download TABLE S1, PDF file, 0.3 MB. Copyright © 2018 Malhotra et al. 2018 Malhotra et al. This content is distributed under the terms of the Creative Commons Attribution 4.0 International license.

**FIG 1:**
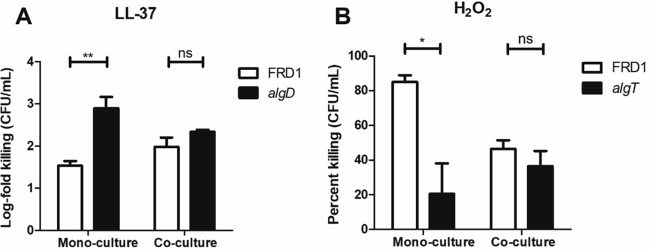
Mucoid and nonmucoid *P. aeruginosa* variants in coculture exhibit enhanced resistance to LL-37 and H_2_O_2_. (A) Monocultures and cocultures of FRD1 (*mucA*) and FRD1 *algD* exposed to 50 µg/ml LL-37 for 1 h. (B) Monocultures and cocultures of FRD1 and FRD1 *algT* exposed to 25 mM H_2_O_2_ for 1 h. Data are represented as log fold (A) or percentage of killing (B) compared to the no-treatment control for each strain/condition. Experiments were performed in duplicate on three independent occasions. Data are presented as mean ± standard error of the mean (SEM). Statistical significance was determined by an unpaired, two-tailed Student’s *t* test. *, *P* < 0.05; **, *P* < 0.01; ns, not significant.

In monoculture, the mucoid strain, FRD1, was significantly more resistant to LL-37 than the nonmucoid strain, FRD1 *algD*, as previously reported (12); however, in coculture, the susceptibilities of both strains to LL-37 were similar, suggesting that FRD1 *algD* was partially rescued from LL-37 by FRD1 (Fig. 1A). Conversely, in monoculture, FRD1 was significantly more susceptible to H_2_O_2_ than FRD1 *algT*; however, in coculture, the susceptibility of FRD1 to H_2_O_2_ was almost identical to that of FRD1 *algT* and significantly reduced compared to the monoculture condition (Fig. 1B). This suggested that the copresence of nonmucoid FRD1 *algT* protected FRD1 from H_2_O_2_ stress. The rationale for using the *algT* revertant (not FRD1 *algD*) in these H_2_O_2_ sensitivity experiments is clarified further below: there was no difference in H_2_O_2_ susceptibilities of FRD1 and FRD1 *algD* in monoculture (see Fig. 3). These results indicated an advantage for mixed-variant, mucoid/nonmucoid populations of *P. aeruginosa* in evading two critical innate immune effectors.

### Alginate is sufficient to protect bacteria from LL-37 killing.

We next sought to define differential mechanisms of immune protection employed by mucoid and nonmucoid variants against LL-37 and H_2_O_2_, respectively. Additionally, we endeavored to understand how these immune evasion strategies might be transferrable between variants in consortia, resulting in advantages observed under coculture conditions (Fig. 1A and B).

**FIG 2:**
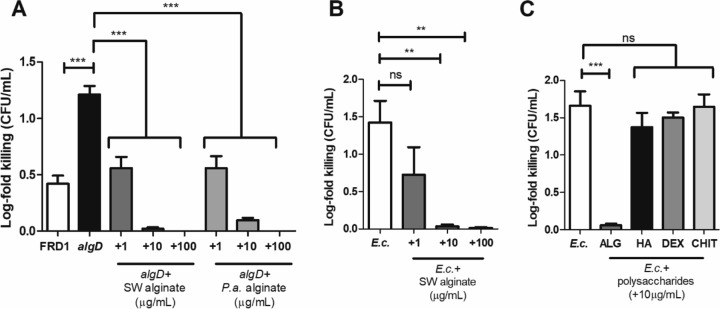
Alginate is sufficient to protect both *P. aeruginosa* and *E. coli* from LL-37-mediated killing. (A) FRD1 (*mucA*), an isogenic FRD1 *algD* strain, or the FRD1 *algD* strain with exogenously added *Pseudomonas aeruginosa* (*P.a.*) or seaweed (SW) alginate at three different concentrations (1, 10, and 100 µg/ml) was treated for 1 h with LL-37 (12.5 µg/ml). Bacteria were plated for CFU per milliliter. Data are presented as log fold killing compared to the no-treatment control. (B) Log fold killing of *E. coli* HB101 by 12.5 µg/ml LL-37 with or without the exogenous addition of SW alginate. (C) Log fold killing of *E. coli* HB101 by 12.5 µg/ml LL-37 with or without the exogenous addition of 10 µg/ml of differentially charged polysaccharides: ALG, SW alginate (anionic); HA, hyaluronic acid (anionic); DEX, dextran (neutral); and CHIT, chitosan (cationic). Experiments were performed in duplicate on three independent occasions. Statistical significance was determined by one-way analysis of variance (ANOVA) followed by Dunnett’s multiple-comparison test. Each condition was compared to either FRD1 *algD* (A) or *E. coli* (B and C) alone. Data are presented as mean ± SEM. **, *P* < 0.01; ***, *P* < 0.001; ns, not significant.

Our previous study identified that LL-37 contributes to mucoid conversion and that mucoid isolates are more resistant to LL-37-mediated killing than *algD* mutants (12). Here, we hypothesized that if the alginate polysaccharide protects against LL-37, then purified alginate added exogenously to nonmucoid *P. aeruginosa* should abrogate LL-37 killing. For these experiments, we used both commercially available, seaweed (SW)- and *P. aeruginosa* (FRD1)-derived alginate. The principal difference between seaweed alginate and *P. aeruginosa* alginate is that only bacterial alginate is O-acetylated (43). Alginate was added exogenously to FRD1 *algD* prior to LL-37 (12.5 µg/ml) exposure. Subsequently, bacteria were plated for colony-forming units (CFU), and log fold killing was calculated (Fig. 2A). Concentrations of LL-37 within CF airway secretions have been measured up to 30 µg/ml (8, 9). Therefore, the concentration of LL-37 used here is within the physiologically relevant range.

As previously reported (12), the nonmucoid *algD* mutant alone was 10-fold more sensitive to LL-37 than the mucoid isolate. However, exogenous addition of both *P. aeruginosa*- and seaweed-derived alginates rescued *algD* from LL-37 killing (Fig. 2A). Alginate in CF sputum is quantitated within the range of 10 to 100 µg/ml (44). Thus, we used 1, 10, and 100 µg/ml of alginate. We observed a dose-dependent reduction in LL-37 killing when adding increasing concentrations of alginate (Fig. 2A). Seaweed alginate was also sufficient to protect *Escherichia coli* (HB101) from LL-37 dose dependently (Fig. 2B). These results suggested that mucoid *P. aeruginosa* resistance to LL-37 is alginate dependent and that alginate as a released product can protect nonmucoid variants and nonpseudomonad species from LL-37.

We further sought to determine whether alginate, an anionic polysaccharide, serves as an electrostatic sink for cationic LL-37. We reasoned if alginate, due to its negative charge, protects bacteria from LL-37 killing, then a different negatively charged polysaccharide may be similarly protective. Three polysaccharides were added to *E. coli* culture prior to LL-37 exposure: hyaluronic acid (anionic), chitosan (cationic), and dextran (neutral). Only alginate protected *E. coli* from LL-37 killing (Fig. 2C). Addition of constituent monosaccharides of alginate, d-mannuronic acid and l-guluronic, acid, alone or in combination, also did not prevent LL-37 killing (see Fig. S2A in the supplemental material). These data suggest alginate’s specific capacity to protect from LL-37 killing is unique as other charged polysaccharides and uronic acid monomers did not confer resistance.

10.1128/mBio.00275-18.3 FIG S2  Uronic acid monomers of alginate do not shield bacteria from LL-37 killing, and addition of calcium does not affect the alginate polymer’s capacity to protect from LL-37. (A) Log fold killing of *E. coli* HB101 (E.c.) by 12.5 µg/ml LL-37 with or without the exogenous addition of 10 µg/ml alginate (ALG) or uronic acids (GA, l-guluronic acid; MA, d-mannuronic acid). (B) Log fold killing of *E. coli* HB101 by 12.5 µg/ml LL-37 with or without the exogenous addition of 10 µg/ml of alginate, which was preincubated with indicated millimolar concentrations of calcium (Ca^2+^). A 2.5 mM Ca^2+^ concentration represents the approximate physiological concentration within the CF airway. Experiments were performed in duplicate on three independent occasions. Statistical significance was measured using one-way ANOVA followed by Dunnett’s multiple-comparison test. Each condition was compared to *E. coli* alone. Data are presented as mean ± SEM. ***, *P* < 0.001; ns, not significant. Download FIG S2, TIF file, 0.8 MB. Copyright © 2018 Malhotra et al. 2018 Malhotra et al. This content is distributed under the terms of the Creative Commons Attribution 4.0 International license.

Divalent cations, specifically calcium (Ca^2+^), change properties of alginate by cross-linking the polysaccharide (45). The Ca^2+^ concentration is also elevated within CF airway secretions (1.9 to 3.0 mM) (46). To investigate whether the presence of Ca^2+^ alters alginate’s capacity to protect against LL-37, we preincubated alginate with Ca^2+^ prior to exogenous addition to bacteria and treatment with LL-37. Physiologically relevant Ca^2+^ concentrations did not affect alginate’s capacity to prevent LL-37 killing (Fig. S2B). In total, in mixed communities, mucoid variants of *P. aeruginosa* protect nonmucoid variants from LL-37 via alginate, independent of Ca^2+^ concentration.

### *algT* mutation confers resistance to H_2_O_2_.

**FIG 3:**
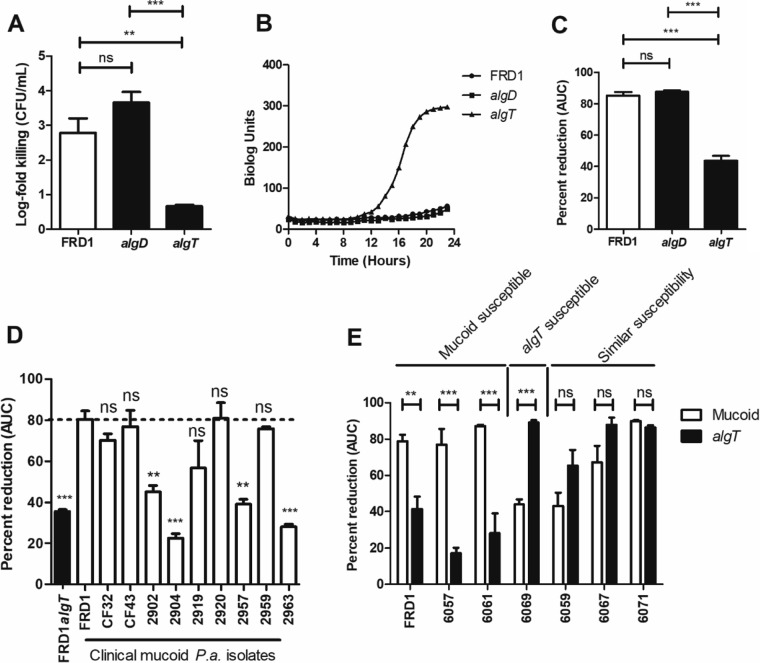
The nonmucoid *algT* revertant is significantly more resistant to hydrogen peroxide than the clinical mucoid isolate, FRD1. (A) One-hour treatment of *P. aeruginosa* strains (FRD1 [*mucA*] and the isogenic *algT* and *algD* mutants) with 50 mM H_2_O_2_, followed by plating for CFU per milliliter. Data are presented as log fold killing relative to the no-treatment condition. (B) Strains grown for 24 h at 37°C in the presence of 25 mM H_2_O_2_ via the OmniLog (Biolog, Inc.) system (C) Kinetic growth curve data from panel B were expressed as a percentage of reduction in AUC relative to the no-treatment condition (Fig. S2A). (D) Clinical mucoid isolates grown for 24 h at 37°C in the presence of 25 mM H_2_O_2_ via the Biolog system. The dotted line represents H_2_O_2_ sensitivity of FRD1. Strains marked as “ns” were as susceptible as FRD1 to H_2_O_2_ (not significant statistically compared to FRD1). (E) Paired mucoid and nonmucoid revertants (*algT* mutants) grown for 24 h at 37°C in the presence of 25 mM H_2_O_2_ via the Biolog system. Experiments were performed in triplicate on three independent occasions. Data are presented as mean ± SEM. Statistical significance was measured by one-way ANOVA followed by Tukey’s multiple-comparison test (A, C, and E) or Dunnett’s multiple-comparison test (D). White bars represent mucoid strains. Black bars represent nonmucoid strains. **, *P* < 0.01; ***, *P* < 0.001.

Given that alginate provided significant protection from LL-37, we anticipated it may also protect against other neutrophil-derived antimicrobials, such as ROS. Previous publications suggested that mucoid *P. aeruginosa* is more resistant to oxidative stress because alginate is a sink for free radicals (47, 48). Therefore, we were surprised at finding no difference in FRD1 and FRD1 *algD* susceptibilities to H_2_O_2_ (Fig. 3A). Paradoxically, FRD1 *algT*, a nonmucoid revertant, was significantly more resistant to H_2_O_2_ killing than the parent strain (Fig. 1B and 3A). Furthermore, in a mixed population, the presence of FRD1 *algT* was sufficient to protect FRD1 from H_2_O_2_ killing (Fig. 1B). The H_2_O_2_ concentrations used here are within a physiologically relevant range (12 to 100 mM) (49).

We used the high-throughput Biolog system (OmniLog; Biolog, Inc.) to investigate susceptibility of mucoid and nonmucoid variants to H_2_O_2_. The Biolog system measures bacterial metabolic activity via a tetrazolium-based dye (50, 51). Untreated FRD1 and nonmucoid (*algT* or *algD*) variants demonstrated similar growth (see Fig. S3A in the supplemental material). However, the *algT* revertant was more resistant to H_2_O_2_ than both FRD1 and FRD1 *algD* (Fig. 3B). Data are represented as the area under the curve (AUC) for each strain treated with H_2_O_2_ as a percentage of the no-treatment condition (Fig. 3C). FRD1 *algT* was also more resistant to hypochlorite (HOCl) than FRD1 (Fig. S3B and C). In total, these findings suggested that H_2_O_2_ susceptibility of mucoid *P. aeruginosa* is relieved by reversion (i.e., *algT* mutation).

10.1128/mBio.00275-18.4 FIG S3  The nonmucoid *algT* revertant is more resistant to hypochlorite (HOCl) than the clinical mucoid isolate, FRD1 (*mucA*). (A) Twenty-four-hour Biolog growth curves of FRD1 (*mucA*), isogenic *algT* and *algD* mutants grown in the presence of medium alone. (B) Twenty-four-hour Biolog growth curves of strains grown in the presence of 11.25 mM HOCl. (C) Kinetic growth curve data from panel B expressed as percentage of reduction in AUC relative to the no-treatment condition. Experiments were performed in triplicate on at least three independent occasions. Statistical significance was measured using one-way ANOVA followed by Dunnett’s multiple-comparison test, wherein each strain was compared to FRD1 (C). Data are presented as mean ± SEM. *, *P* < 0.05; ns, not significant. Download FIG S3, TIF file, 0.4 MB. Copyright © 2018 Malhotra et al. 2018 Malhotra et al. This content is distributed under the terms of the Creative Commons Attribution 4.0 International license.

### Clinical mucoid *P. aeruginosa* isolates demonstrate susceptibility to H_2_O_2_.

Given that mucoidy did not protect against H_2_O_2_ killing, we sought to investigate whether this phenotype was specific to FRD1 or generalizable across multiple clinical mucoid strains. We first tested the H_2_O_2_ susceptibilities of nine additional mucoid CF isolates. We found that 5/9 strains tested exhibited H_2_O_2_ sensitivity similar to that of FRD1, while 4/9 did not (Fig. 3D). Given that each isolate tested overproduced alginate, the sensitivity differences observed between these two groups is unlikely to be attributable to alginate production and reveals that the H_2_O_2_ sensitivity of mucoid strains may not be specific to FRD1.

We subsequently tested H_2_O_2_ susceptibility of multiple isogenic mucoid and *algT* revertant pairs of *P. aeruginosa* strains (Fig. 3E). Mucoid strains in this screen represent clinical isolates from CF patients (33); spontaneous *algT* revertants were isolated either from patients or via growth of the mucoid variant *in vitro* (33). Although not all strains tested behaved as FRD1 or FRD1 *algT*, two pairs did recapitulate the previously observed phenotype, wherein the mucoid isolate was more susceptible to H_2_O_2_. While differences in H_2_O_2_ sensitivities among clinical isolates were intriguing, these were not attributable to mucoidy alone. All mucoid isolates tested overproduced alginate (see Fig. S4A in the supplemental material). There was no correlation between H_2_O_2_ susceptibilities and alginate production (Fig. S4B). These results supported our previous findings suggesting that mucoidy alone was insufficient to protect *P. aeruginosa* from H_2_O_2_ stress.

10.1128/mBio.00275-18.5 FIG S4  Alginate production by paired mucoid and *algT* isolates does not correlate with H_2_O_2_ susceptibility phenotype. (A) Quantification of alginate production by paired mucoid and *algT* revertants via carbazole assay. The limit of detection was set equal to zero. (B) H_2_O_2_ susceptibility of each paired mucoid and *algT* strain plotted versus alginate production by each strain. Lines of best fit for both mucoid and *algT* strains are shown with *r*^2^ values of 0.0008 and 0.002, respectively. Data are presented as mean ± SEM. Alginate quantitation of all strains was performed in duplicate on three independent occasions. Download FIG S4, TIF file, 0.8 MB. Copyright © 2018 Malhotra et al. 2018 Malhotra et al. This content is distributed under the terms of the Creative Commons Attribution 4.0 International license.

### *algT* mutant supernatants protect FRD1 from H_2_O_2_ stress via KatA.

**FIG 4:**
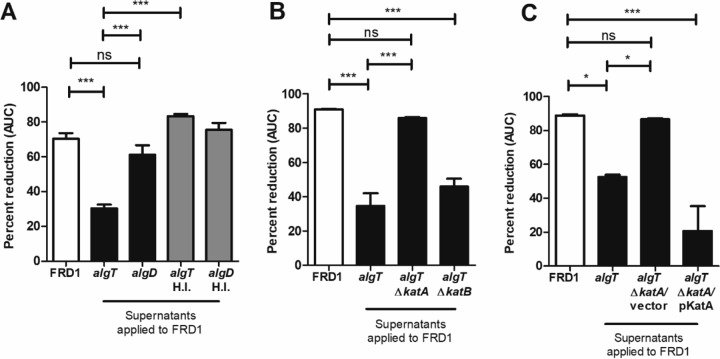
Supernatants derived from the *algT* revertant are sufficient to protect mucoid *P. aeruginosa* from H_2_O_2_ killing via KatA. (A) FRD1 (*mucA*) was resuspended in cell-free supernatants derived from the *algT* or *algD* strain prior to growth for 24 h in the presence of 25 mM H_2_O_2_ via the Biolog system. Supernatants were collected and filter sterilized from strains after overnight growth. Heat-inactivated supernatants, incubated at 80°C for 30 min, are designated “HI.” Growth curve data are shown as percentage of reduction in AUC relative to the no-treatment condition. (B and C) FRD1 resuspended in supernatants derived from *algT*, *algT* Δ*katA*, and *algT* Δ*katB* mutants (B) or *algT* Δ*katA*/vector and *algT* ΔkatA/pKatA (C) prior to 24 h of growth in the presence of 25 mM H_2_O_2_ via the Biolog system. Experiments were performed in triplicate on three independent occasions. Data are presented as mean ± SEM. Statistical significance was measured by one-way ANOVA followed by Tukey’s multiple-comparison test (A to C). *, *P* < 0.05; ***, *P* < 0.001; ns, not significant.

Given that three pairs of mucoid and *algT* revertants tested (including FRD1) showed *algT* mutants were more resistant to H_2_O_2_, we sought to elucidate the mechanism underlying this phenotype. In coculture, FRD1 *algT* protected FRD1 from H_2_O_2_ killing (Fig. 1B), suggesting secretion of a soluble antioxidant. To test this experimentally, we filter sterilized supernatants from stationary-phase cultures of FRD1 and *algT* and *algD* mutant strains. FRD1 was resuspended in these supernatants, and H_2_O_2_ susceptibility was tested. Only supernatants from FRD1 *algT* significantly protected FRD1 from H_2_O_2_ killing (Fig. 4A). Supernatants derived from FRD1 *algD* or FRD1 itself did not protect from H_2_O_2_. Heat inactivation of *algT* supernatants abrogated protection, suggesting a heat-labile protein was responsible for H_2_O_2_ resistance (Fig. 4A).

Two catalases produced by *P. aeruginosa*, KatA and KatB, play a vital role in protection against H_2_O_2_ stress (31, 52–54). Supernatants derived from an *algT* Δ*katA* mutant abrogated protection of FRD1 against H_2_O_2_ compared to the parent *algT* revertant. In contrast, supernatants from the *algT* Δ*katB* mutant still protected FRD1 from H_2_O_2_ (Fig. 4B). Protection from H_2_O_2_ was restored in the *algT* Δ*katA* mutant by expression of *katA* in *trans* (Fig. 4C). These data suggested the revertant protected mucoid *P. aeruginosa* from H_2_O_2_ via KatA, which was released into the extracellular milieu.

### AlgT indirectly represses *katA* transcription via AlgR.

**FIG 5:**
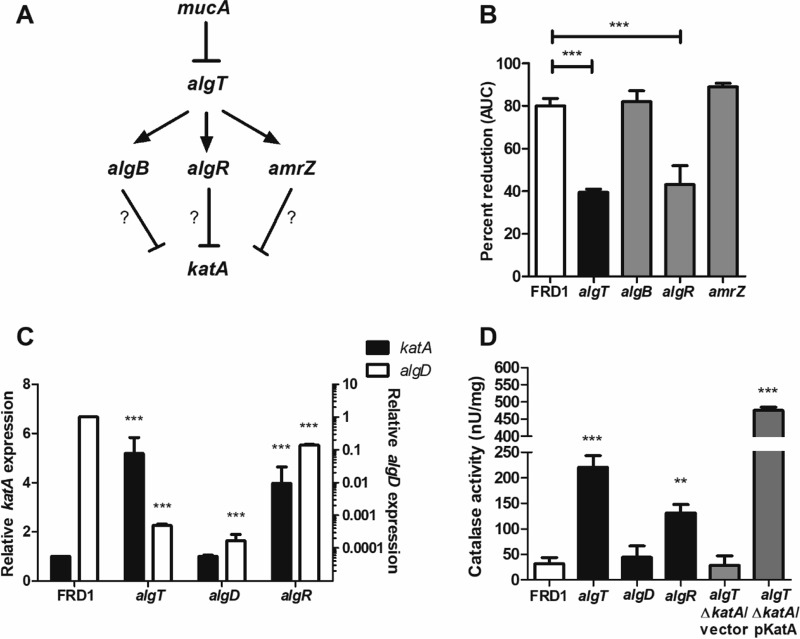
*katA* transcription is negatively regulated by AlgT, via AlgR. (A) Potential pathway for regulation of *katA* transcription by AlgT through one of three downstream transcription factors: AlgB, AlgR, and AmrZ. (B) FRD1 (*mucA*) and the isogenic *algT*, *algB*, *algR*, and *amrZ* mutants were grown for 24 h in the presence of 25 mM H_2_O_2_ via the Biolog system. Data are plotted as the percentage of AUC relative to the no-treatment condition. (C) *katA* and *algD* mRNA levels quantitated by qRT-PCR, relative to FRD1. (D) Quantitation of catalase protein activity within cell-free supernatants of *P. aeruginosa* strains using the BioVision catalase activity colorimetric assay. Experiments were performed in triplicate (B and C) or duplicate (D) on at least three independent occasions. Statistical significance was measured using one-way ANOVA followed by Tukey’s multiple-comparison test (B and C) or Dunnett’s multiple-comparison test (D), wherein each strain was compared to FRD1. Data are presented as mean ± SEM. **, *P* < 0.01; ***, *P* < 0.001; ns, not significant.

Given that mutation of *algT* provided enhanced resistance to H_2_O_2_ through KatA, we proposed *algT* may act as a repressor of *katA* transcription. Alternative sigma factors typically suppress gene transcription indirectly, via downstream transcription factors (55–57). In the alginate system, three main transcription factors lie downstream of AlgT: AlgB, AlgR, and AmrZ (Fig. 5A) (19, 20). Each of these factors (including AlgT) is necessary for expression of the alginate biosynthetic operon. We reasoned that if these factors directly repressed *katA* transcription, then mutation of genes that encode these factors in a *mucA* background should result in enhanced resistance to H_2_O_2_.

Indeed, *algR* mutation resulted in enhanced resistance to H_2_O_2_ compared to FRD1, while *algB* and *amrZ* mutants remained H_2_O_2_ sensitive (Fig. 5B). Consistent with this finding, *katA* transcription is elevated in both *algT* and *algR* mutants relative to FRD1 (Fig. 5C). *katA* transcription was unchanged in the *algD* mutant compared to FRD1. We also measured *algD* transcript as an additional control in this experiment. Consistent with previously published work (19, 57), mutation of *algT*, *algR*, or *algD* results in significant reduction of *algD* transcription relative to the *mucA* isolate.

Catalase protein activity was quantitated by a commercially available catalase enzyme activity kit. Catalase activity was significantly higher in supernatants of *algT* and *algR* mutants relative to FRD1 (Fig. 5D), corresponding with the elevated *katA* transcription (Fig. 5C). Supernatants from the *algT* Δ*katA* strain demonstrated significant loss of catalase activity; *katA* complementation restored activity (Fig. 5D). In total, these data suggested AlgT is an indirect repressor of *katA* transcription via AlgR.

### Extracellular release of KatA is dependent on *lys-*mediated cell lysis.

Previous publications had shown KatA within the periplasmic space and predicted KatA is released via cell lysis (53). However, a clear mechanism linking autolysis in *P. aeruginosa* and KatA release was not elucidated.

Recently, a bacteriophage endolysin encoded by *lys* (PA0629), found within the R- and F-pyocin gene cluster, was shown to mediate explosive cell lysis and extracellular DNA (eDNA) release in *P. aeruginosa* (58). Here, we sought to determine whether *lys* also has a role in KatA release. Two previously published studies supported this investigation. First, the expression of *lys* was elevated in response to H_2_O_2_ exposure in *P. aeruginosa*, suggesting that cell lysis may be an adaptive response against H_2_O_2_ stress (59). Furthermore, in our previous work comparing the transcriptomes of FRD1 and an isogenic *algT* mutant, *lys* expression was upregulated in the *algT* mutant, suggesting that cell lysis in FRD1 *algT* might contribute to H_2_O_2_ resistance (60).

**FIG 6:**
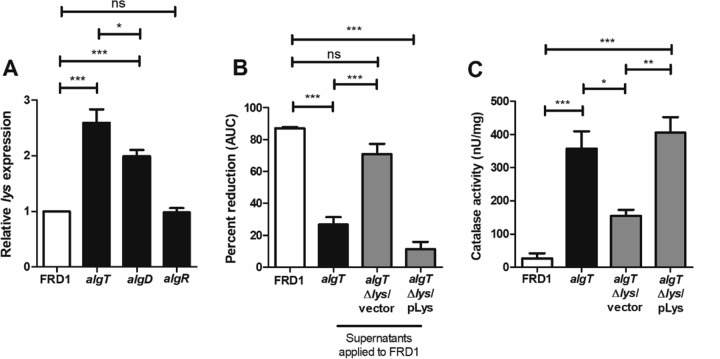
Deletion of *lys* abrogates catalase release in *algT* revertants. (A) *lys* mRNA levels quantitated by qRT-PCR relative to FRD1. (B) Percentage of reduction in AUC for FRD1 resuspended in supernatants from the *algT*, *algT* Δ*lys*, and *algT* Δ*lys*/pLys strains prior to 24 h of growth in the presence of H_2_O_2_ by the Biolog system. (C) Quantitation of catalase protein activity within cell-free supernatants of *P. aeruginosa* strains using the BioVision catalase activity colorimetric assay. Experiments were performed in triplicate (A and B) or duplicate (C) on at least three independent occasions. Statistical significance was measured using one-way ANOVA followed by Tukey’s multiple-comparison test (A to C). Data are presented as mean ± SEM. *, *P* < 0.05; **, *P* < 0.01; ***, *P* < 0.001; ns, not significant.

Consistent with previous findings, *lys* mRNA was elevated in FRD1 *algT* compared to FRD1 (Fig. 6A). Surprisingly, *lys* transcription was also elevated in the *algD* mutant but not in the *algR* mutant. These results suggested that *algT* mutation likely derepresses *lys* transcription via an *algR*-independent pathway. Subsequently, we generated both a *lys* mutant and its complement in the FRD1 *algT* background. To validate that the Δ*lys* mutant exhibited reduced cell lysis, we measured eDNA present within the supernatants of our strains as a surrogate for cell lysis. We derived supernatants from FRD1 wild-type and *algT*, *algT* Δ*lys*, and *algT*Δ *lys*/p*lys* strains, and similar to a previously published approach (61), these supernatants were analyzed by agarose gel electrophoresis. A high-molecular-weight band was observed for each strain (see Fig. S5A in the supplemental material), suggestive of eDNA. Quantification of band intensity revealed that the *algT* revertant underwent more cell lysis (i.e., showed greater eDNA release) than FRD1 (Fig. S5B). Furthermore, the *algT* Δ*lys* strain showed reduced cell lysis, which was restored by complementation (Fig. S5B).

10.1128/mBio.00275-18.6 FIG S5  Deletion of *lys* reduces eDNA release by *algT* revertants. (A) eDNA visualized via electrophoresis of cell-free supernatants from FRD1 (*mucA*) or *algT*, *algT* Δ*lys*, and *algT* Δ*lys*/pLys mutants. eDNA was observed as a high-molecular-weight band (>3,000 bp). MWM, molecular weight marker. One representative gel image is shown. (B) Quantification of eDNA band intensity using the densitometry plugin in ImageJ. Densitometry results represent the average from three independent experiments. Statistical significance was measured using one-way ANOVA followed by Tukey’s multiple-comparison test. Data are presented as mean ± SEM. ***, *P* < 0.001; ns, not significant. Download FIG S5, TIF file, 1.9 MB. Copyright © 2018 Malhotra et al. 2018 Malhotra et al. This content is distributed under the terms of the Creative Commons Attribution 4.0 International license.

Next, we wanted to ascertain whether the *algT* Δ*lys* strain also released less catalase than its parent strain. We hypothesized that if deletion of *lys* resulted in reduced catalase release, then supernatants from the *algT* Δ*lys* strain would be less effective in protecting FRD1 from H_2_O_2_ stress. Indeed, FRD1 resuspended in supernatants derived from the *algT* Δ*lys* strain was significantly more susceptible to H_2_O_2_ than when resuspended in supernatants from the *algT* or complemented Δ*lys* mutant (Fig. 6B). Correspondingly, there was reduced catalase activity in cell-free supernatants of the *algT* Δ*lys* mutant as well, which was restored by complementation (Fig. 6C). To our knowledge, these results for the first time link a specific mechanism for cell lysis and extracellular release of *P. aeruginosa* catalase.

## DISCUSSION

**FIG 7:**
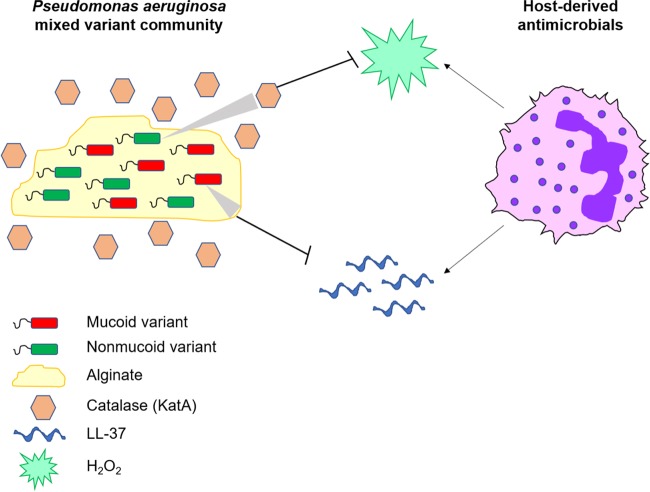
Model of mixed communities of mucoid variants with nonmucoid revertants evading host-derived antimicrobials within the CF lung. Mucoid *P. aeruginosa* variants protect mixed-variant populations from LL-37 via alginate production. Nonmucoid revertants protect the mixed population by releasing catalase (KatA), which neutralizes H_2_O_2_.

Mixed communities of mucoid and nonmucoid *P. aeruginosa* strains are frequently isolated from chronically infected CF patients (33–37). Given the well-understood recalcitrance of the mucoid phenotype to clearance by antibiotics and immune cells, the selective benefit of nonmucoid variants within these mixed communities has not been elucidated. Here, we have shown that mixed-variant *P. aeruginosa* populations have an advantage in evading two critical immune effectors: LL-37 and H_2_O_2_. This benefit of coexistence is mediated by production and sharing of two public goods: mucoid variants overproduce alginate to protect against LL-37, and nonmucoid *algT* revertants overproduce catalase (KatA) to neutralize H_2_O_2_ (Fig. 7).

We had previously shown that LL-37 contributes to mucoid conversion in *P. aeruginosa* and that mucoid isolates were significantly more resistant than isogenic nonmucoid variants to this peptide (12). Here, we demonstrated that the addition of alginate exogenously to a nonmucoid *algD* mutant was sufficient to rescue this strain from LL-37 killing. Furthermore, alginate was sufficient to protect *E. coli* from LL-37-killing. This substantiates previous work showing alginate added exogenously to other Gram-negative pathogens (e.g., *Klebsiella pneumoniae*) can provide resistance to AMPs such as polymyxin B and α-defensin-1 (HNP-1) (62). The presence of free alginate in CF sputum and in association with lung mucosa is well documented (34, 35, 44). Our results, taken together with past findings, suggest that within the extracellular milieu of the CF airway, alginate protects bacteria from AMPs, independent of genera/species.

In investigating whether the polyanionic charge of alginate plays a role in LL-37 evasion, we found an uncharged polysaccharide (dextran) and a cationic polysaccharide (chitosan) did not protect from LL-37. Hyaluronic acid, a negatively charged polysaccharide, also did not shield from LL-37 killing. It is tempting to conclude based on these data that a unique property of alginate, independent of its anionic charge, is responsible for LL-37 resistance. Nonetheless, previous studies demonstrate that the primary mode of interaction between alginate and AMPs is likely electrostatic in nature: alginate binds AMPs via ionic interactions, which induce peptide α-helix formation and aggregation, inhibiting AMP bactericidal function (63, 64). In one study, the amount of negative charge per chemical repeating unit within polyanionic polysaccharides correlated with protection against LL-37 killing (65). Hyaluronic acid has half the negative charge density of alginate (66). Thus, the greater negative charge of alginate may explain how it binds and protects from LL-37 more effectively than does hyaluronic acid.

Although *P. aeruginosa* susceptibility to LL-37 was alginate dependent, H_2_O_2_ sensitivity was significantly reduced in a nonmucoid *algT* revertant compared to the mucoid parent. These results were surprising for two reasons: First, both LL-37 and H_2_O_2_ are known to induce mucoid conversion, and as is true for LL-37, we expected mucoid variants to be resistant to their own pathoadaptive triggers, including ROS (11, 12). Second, two often-cited publications illustrate how alginate acts as a sink for ROS (47, 48). Both publications demonstrate that addition of alginate to stimulated phagocytes reduces detection of ROS without affecting viability of the immune cells. However, another publication directly contradicted these results, showing that addition of alginate to neutrophils enhances oxidative burst (44). In support of our findings, Brown et al. found that catalase protein activity was lower in FRD1 than that in an *algT* mutant, suggesting H_2_O_2_ susceptibility of mucoid isolates may be attributable to catalase, not to alginate (31).

Nevertheless, to investigate whether mucoidy is sufficient to protect against ROS and to rule out that FRD1 is unique in its susceptibility to H_2_O_2_, we screened the H_2_O_2_ susceptibilities of a panel of clinical mucoid *P. aeruginosa* isolates as well as isogenic pairs of mucoid *algT* revertants. These data reassured us that the FRD1 or FRD1 *algT* phenotype was represented in multiple, but not all *P. aeruginosa* isolates from CF patients. However, we did observe some variability among clinical isolates: in the second screen, some mucoid *algT* pairs exhibited no difference in H_2_O_2_ susceptibility, and in one case, the mucoid isolate was more resistant to H_2_O_2_ than the *algT* revertant. Differences in alginate production among these strains did not account for the different H_2_O_2_ susceptibility phenotypes. One possible explanation for these differences could be the *algT* revertants have distinct *algT* mutations, which perturb sigma factor function differently, resulting in variable H_2_O_2_ sensitivity phenotypes. Future work will seek to test this hypothesis through sequencing of the *algT* locus across multiple nonmucoid revertants to determine whether specific *algT* mutations cluster with H_2_O_2_ susceptibility phenotypes.

In focusing our mechanistic studies here on FRD1, we found that supernatants from FRD1 *algT* protected the mucoid strain from H_2_O_2_ stress in a *katA*-dependent manner. In *P. aeruginosa*, *katA* encodes a constitutively expressed catalase, whereas *katB* expression is induced upon exposure to H_2_O_2_ (31). Both catalases are localized in different cellular compartments: while KatB is restricted to the cytosol, KatA is found in both the cytosol and periplasm, suggesting KatA may be secreted or released (31). This may explain why only supernatants derived from the *algT* Δ*katA* strain, but not those from the *algT* Δ*katB* strain, showed complete loss of protection from H_2_O_2_. These data corroborate previous findings showing KatA (but not KatB) in the extracellular milieu of *P. aeruginosa* (53, 54).

We further demonstrated that *katA* transcription is negatively regulated by AlgT, via AlgR. Although an aforementioned study had shown that catalase protein activity is higher in FRD1 *algT* than in FRD1, the H_2_O_2_ susceptibility of these strains and a pathway for *algT*-dependent transcriptional repression of *katA* were not investigated (31). Furthermore, Lizewski et al. published that an *algR* mutant in a non-*mucA* strain background (PAO1) exhibits greater resistance to H_2_O_2_ than the wild-type strain (67). In later work, via microarray, they also showed that *katA* transcription is elevated (1.8-fold) in the *algR* mutant compared to PAO1, without attributing this to possible AlgT-dependent effects (68). Our findings here connect the prior work by Brown et al. and Lizewski et al. by providing evidence for AlgT repression of *katA* transcription via AlgR, thus elucidating a specific mechanism for enhanced H_2_O_2_ tolerance of *algT* revertants.

We also linked *lys*-mediated autolysis to the release of catalase and evasion of H_2_O_2_ killing in *P. aeruginosa*. While *lys* expression was elevated in FRD1 *algT*, it was not increased in the *algR* mutant, despite *katA* expression, catalase protein activity, and H_2_O_2_ resistance being elevated in both strains. This finding suggests two possibilities: either *lys* transcriptional regulation is *algT* dependent and *algR* independent (i.e., *lys* is directly repressed by a different transcription factor downstream of AlgT), or *algR* mutants exhibit autolysis in a *lys*-independent manner, explaining the detection of catalase in cell-free supernatants of the *algR* mutant, albeit less than in the *algT* mutant. Examining the validity of these hypotheses will be the subject of future work.

Given the long-term persistence of *P. aeruginosa* mucoid variants within the CF lung, it seems logical that H_2_O_2_-susceptible mucoid variants may be shielded and sustained by the presence of coinfecting nonmucoid variants. Moreover, ROS such as H_2_O_2_ (and HOCl) may represent important host factors that select for revertants within the CF lung. However, these data also begged the question of whether nonmucoid progenitor strains (*mucA*^*+*^ and *algT*^*+*^) of *P. aeruginosa*, wherein wild-type MucA would be predicted to antagonize AlgT activity, are equally as resistant to H_2_O_2_ as nonmucoid revertants (*mucA* and *algT* mutants) (Fig. S1). Although the progenitor of FRD1 has never been isolated, we generated a “pseudoprogenitor” via complementation of *mucA* in FRD1 (FRD1/pMucA). Indeed, both the nonmucoid revertant and the progenitor were more resistant to H_2_O_2_ than the mucoid variant (see Fig. S6A in the supplemental material). Furthermore, supernatants derived from both the progenitor and revertant protected FRD1 from H_2_O_2_ stress (Fig. S6B). These data suggest that H_2_O_2_ resistance depends on inactivation of AlgT, and both nonmucoid progenitors and *algT* revertants could play a role in evasion of H_2_O_2_ within mixed-variant communities.

10.1128/mBio.00275-18.7 FIG S6  Complementation of *mucA* restores H_2_O_2_ resistance of FRD1. (A) FRD1 (*mucA*) and the isogenic *algT* and *mucA*/pMucA strains were grown for 24 h in the presence of 25 mM H_2_O_2_ via the Biolog system. Data are plotted as a percentage of the AUC relative to the no-treatment condition. (B) *mucA* cells resuspended in supernatants derived from *algT* and *mucA*/pMucA cells prior to 24 h of growth in the presence of 25 mM H_2_O_2_ by the Biolog system. Experiments were performed in triplicate on at least three independent occasions. Statistical significance was measured using one-way ANOVA followed by Tukey’s multiple-comparison test. Data are presented as mean ± SEM. ***, *P* < 0.001; ns, not significant. Download FIG S6, TIF file, 0.5 MB. Copyright © 2018 Malhotra et al. 2018 Malhotra et al. This content is distributed under the terms of the Creative Commons Attribution 4.0 International license.

All experiments in this study were performed under *in vitro* conditions with planktonic cultures of bacteria, wherein mucoid and nonmucoid variants were mixed in a 1:1 ratio. As such, we acknowledge that the advantages of mucoid and nonmucoid communities demonstrated here in evading an antimicrobial peptide and ROS may only capture part of the total benefits realized in these populations *in vivo*. Nevertheless, the remarkable capacity of *P. aeruginosa* to adapt to stress via the acquisition of stable mutations is well established (69–71). Multiple variants of *P. aeruginosa* with different colony morphotypes have been found to coexist within the CF lung, including mucoid variants, nonmucoid revertants, and small-colony variants (SCVs), among others (72). The selective advantage of these mixed *P. aeruginosa* populations in evading the host response illustrates the insurance hypothesis: an ecologic principle postulating, the fitness of a community to withstand stress is enhanced by genotypic/phenotypic diversity (73, 74). The CF lung represents an environment that changes over time, through the age of the patient, stage of disease, coinfecting microbes, and treatment with various therapeutics (75–79). The genotypic and functional diversification of *P. aeruginosa* likely contributes to adaptation under these stressful conditions, enabling long-term colonization of the CF airway. This study argues for continued examination of mixed-variant *P. aeruginosa* communities as significant contributors to disease pathology.

## MATERIALS AND METHODS

### Strains and growth conditions.

All *P. aeruginosa* strains were maintained on *Pseudomonas* isolation agar (PIA), followed by growth in Luria broth with no salt (LBNS). *E. coli* strains were maintained on Luria agar (LA), followed by growth in Luria broth (LB). All gene mutations were made as previously described by overlap extension PCR (80). For plasmid maintenance, 100 µg/ml (*E. coli*) or 300 µg/ml (*P. aeruginosa*) ampicillin was added to the media. In coculture experiments, parental and derivative strains were selected with 150 µg/ml streptomycin or 100 µg/ml rifampin (RIF), respectively. Arabinose (0.2%) was used to induce expression of genes from the pHERD20T arabinose-inducible vector. All primers, plasmids, and strains used are delineated in Text S1 and Table S1 in the supplemental material).

10.1128/mBio.00275-18.1 TEXT S1  Supplemental materials and methods. Download TEXT S1, PDF file, 0.4 MB. Copyright © 2018 Malhotra et al. 2018 Malhotra et al. This content is distributed under the terms of the Creative Commons Attribution 4.0 International license.

### One-hour bacterial killing.

Bacterial strains were grown to mid-exponential phase (approximately an optical density at 600 nm [OD_600_] of ~0.5 or 2 × 10^8^ CFU/ml). For H_2_O_2_ killing assays, bacteria were mixed 1:1 with H_2_O_2_ diluted in LBNS and incubated for 1 h at 37°C, followed by plating for CFU per milliliter on PIA. Data were expressed as log fold killing relative to the no-treatment condition. For the LL-37 (Sigma) killing assays, bacteria at the exponential phase were pelleted and resuspended in sodium phosphate buffer (SPB) at pH 6.4. Bacteria were mixed 1:1 with LL-37 diluted in SPB and incubated 1 h at 37°C, followed by plating for CFU per milliliter on either PIA or LA. For experiments in which mono- or polysaccharides were added exogenously, seaweed alginate (Sigma), hyaluronic acid (Sigma), dextran (Sigma), chitosan (MP Biomedicals), mannuronic acid (Sigma), and guluronic acid (Carbosynth, Compton, United Kingdom) were obtained commercially. *P. aeruginosa* alginate was purified as described below.

### Monoculture versus coculture bacterial killing.

Monoculture versus coculture killing assays were performed identically to the 1-h killing assay protocol described above. Under the coculture conditions, strains were mixed 1:1 prior to exposure to either LL-37 or H_2_O_2_. Cultures were plated on selective media to determine CFU.

### Alginate purification and quantitation.

*P. aeruginosa* alginate was purified and quantitated as previously described (57). Additional details are provided in Text S1.

### Biolog growth inhibition and supernatant protection assays.

Overnight bacterial cultures were diluted to an OD_600_ of 0.24. To generate a master mix for each bacterial strain, 150 µl of bacterial culture was added to 850 µl LBNS with 12 µl of Biolog dye A. Fifty microliters was transferred to a Biolog 96-well plate in triplicate. Then, 50 µl of H_2_O_2_ (diluted in LBNS at the desired concentration) or LBNS alone was added to each well containing bacteria. Plates were placed in the OmniLog incubator at 37°C for 24 h. The output of the system is growth curves, which can be plotted (as Biolog units versus time in hours) using Biolog’s kinetic software (OL_FM_12) package. Data are also presented as the area under the curve (AUC), which was generated from Biolog’s parametric (OL_PR_12) software. The percentage of reduction (AUC) was calculated by taking the AUC in the presence of H_2_O_2_ as a percentage of the no-treatment condition.

To assess if bacterial supernatants from various strains were sufficient to protect FRD1 from H_2_O_2_ stress, overnight bacterial cultures were pelleted. Supernatants were collected and filter sterilized. Overnight FRD1 culture was diluted in fresh medium to an OD_600_ of 0.24. Five hundred microliters was pelleted and resuspended with 500 μl of supernatant from desired strains. H_2_O_2_ susceptibility was then assayed by Biolog as detailed above.

### qRT-PCR.

Quantitative reverse transcriptase PCR (qRT-PCR) was performed to measure mRNA levels of desired genes in bacterial strains of interest as described previously (81). Additional details are provided in Text S1.

### Catalase activity assays.

To measure catalase protein activity in the cell-free supernatants of *P. aeruginosa* strains, a commercially available kit (BioVision catalase activity colorimetric/fluorometric assay) was used per the manufacturer’s instructions. Additional details are provided in Text S1.

### Statistical analysis.

Statistical analyses were performed using GraphPad Prism v.5 (GraphPad Software, Inc.). Statistical significance was determined using a *P* value of <0.05. Three biological replicates were performed in triplicate for all experiments unless otherwise specified.
