# Supplementary figures and images for: Mixed Communities of Mucoid and Nonmucoid Pseudomonas aeruginosa Exhibit Enhanced Resistance to Host Antimicrobials
Source: mBio. 2018 Mar 27;9(2):e00275-18. doi: 10.1128/mBio.00275-18 (PMC5874919; doi:10.1128/mBio.00275-18)

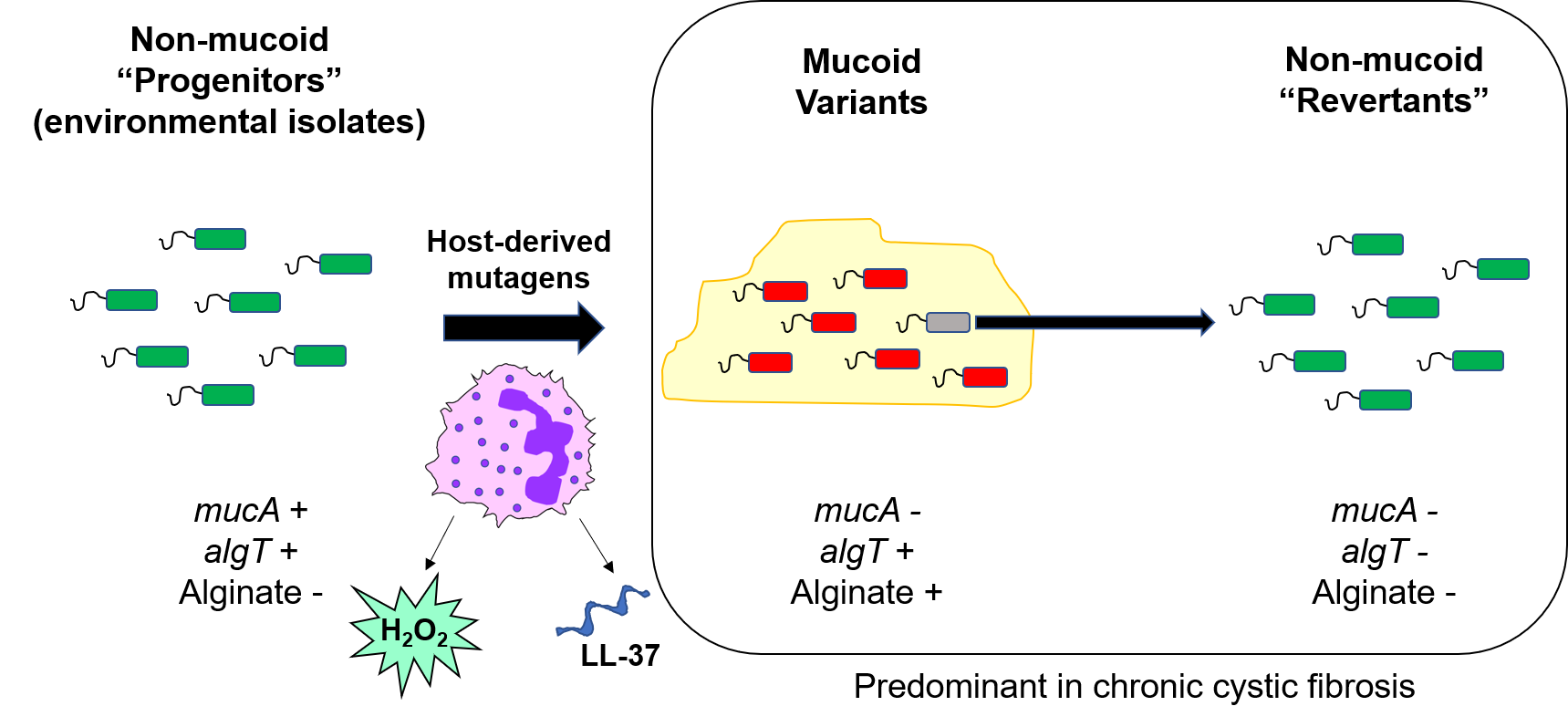

Supplement: FIG S1 [file mbo002183774sf1.tif]

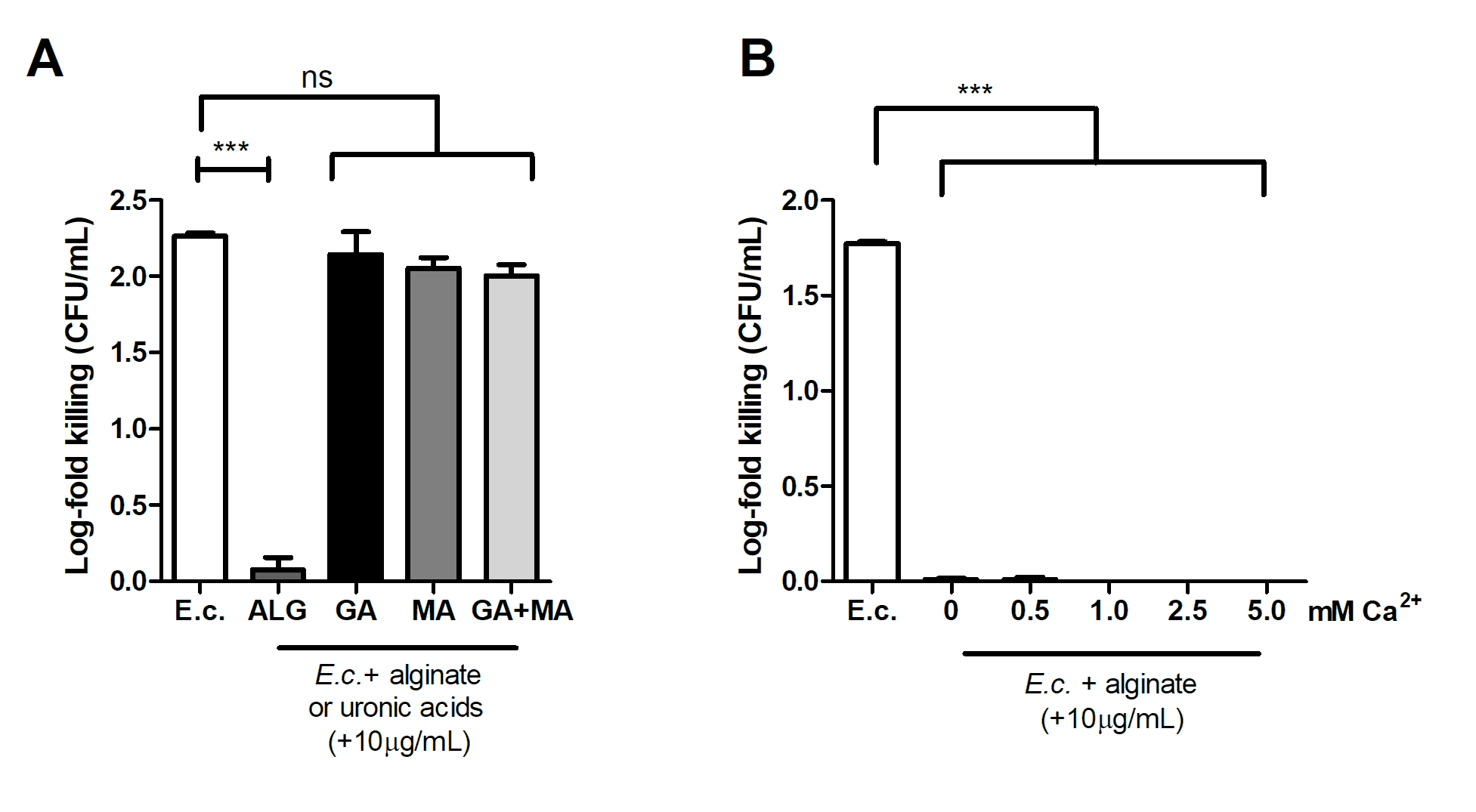

Supplement: FIG S2 [file mbo002183774sf2.tif]

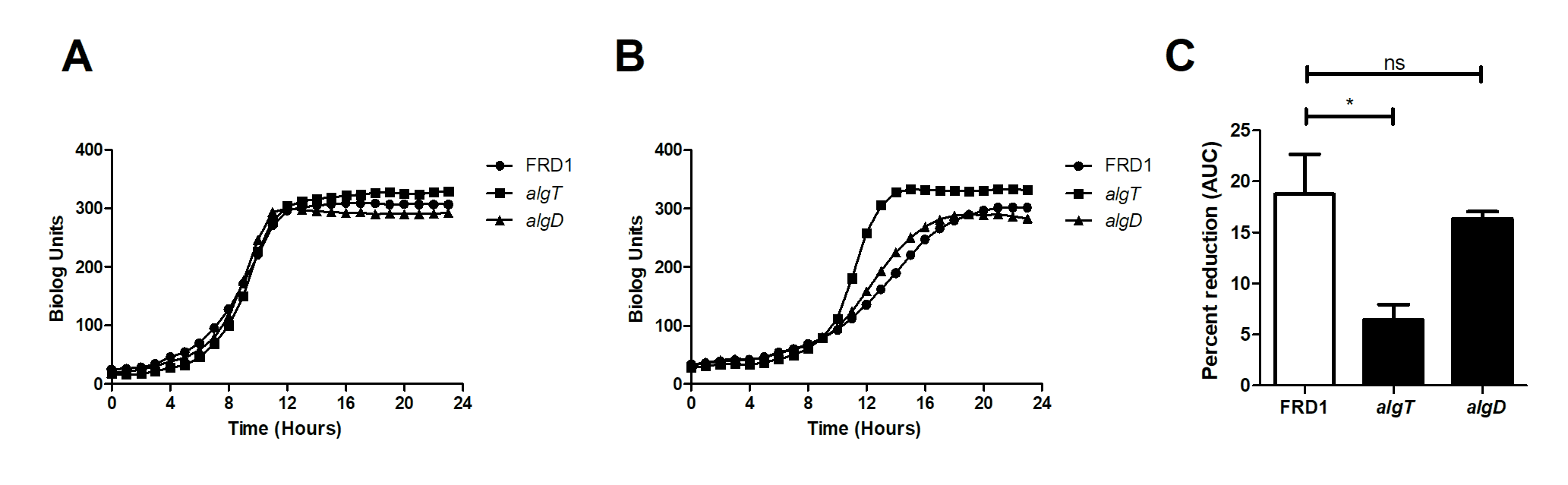

Supplement: FIG S3 [file mbo002183774sf3.tif]

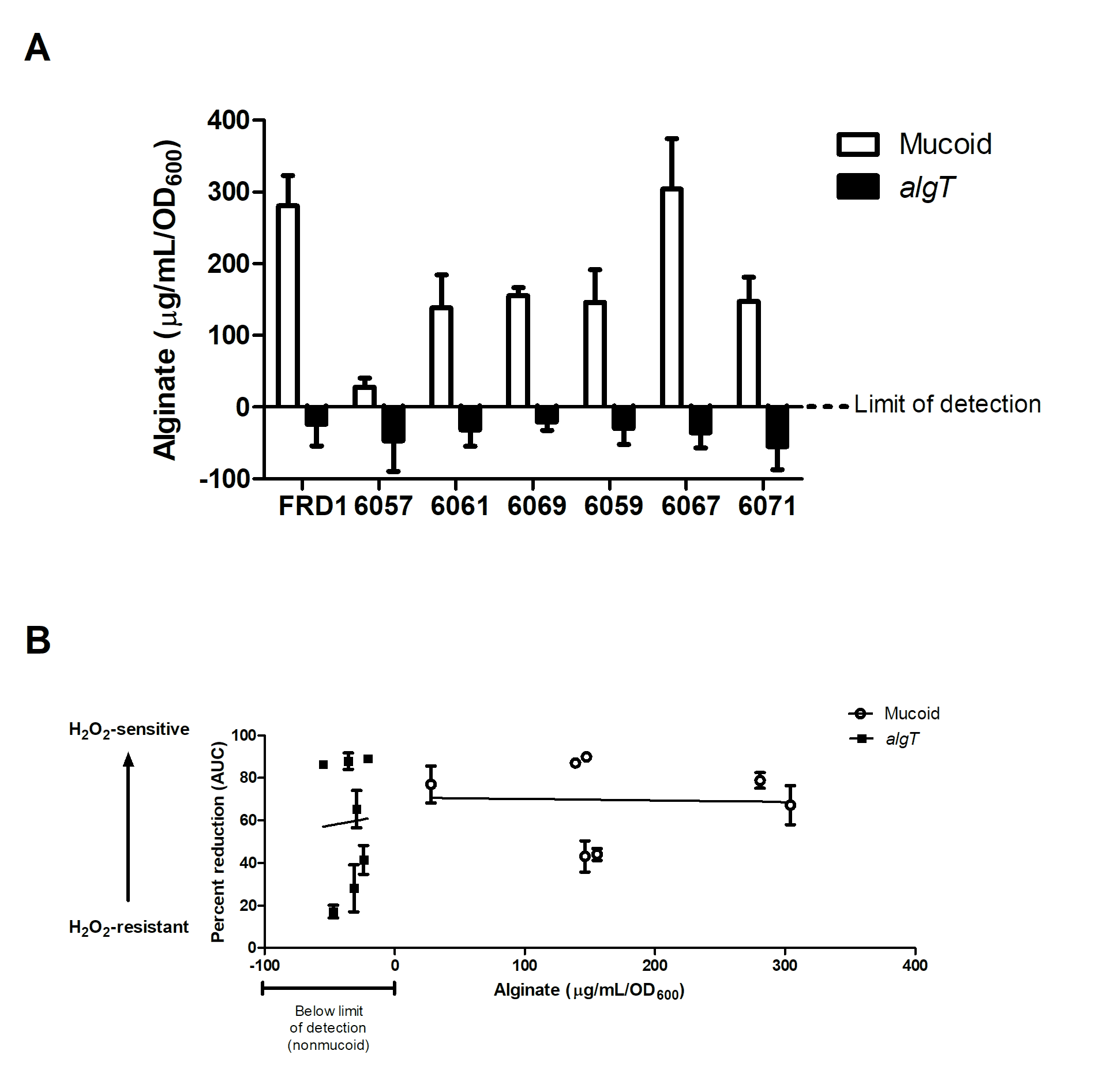

Supplement: FIG S4 [file mbo002183774sf4.tif]

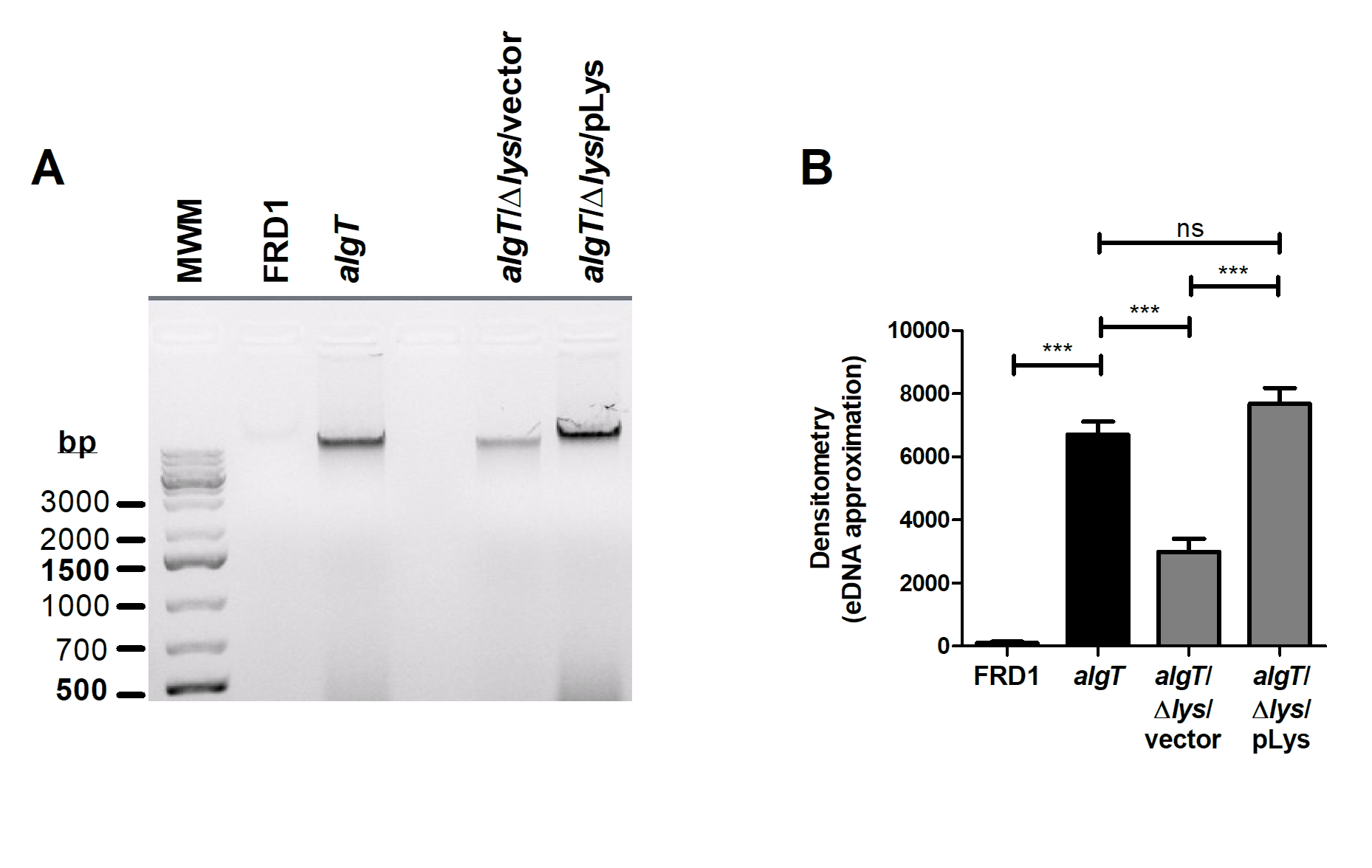

Supplement: FIG S5 [file mbo002183774sf5.tif]

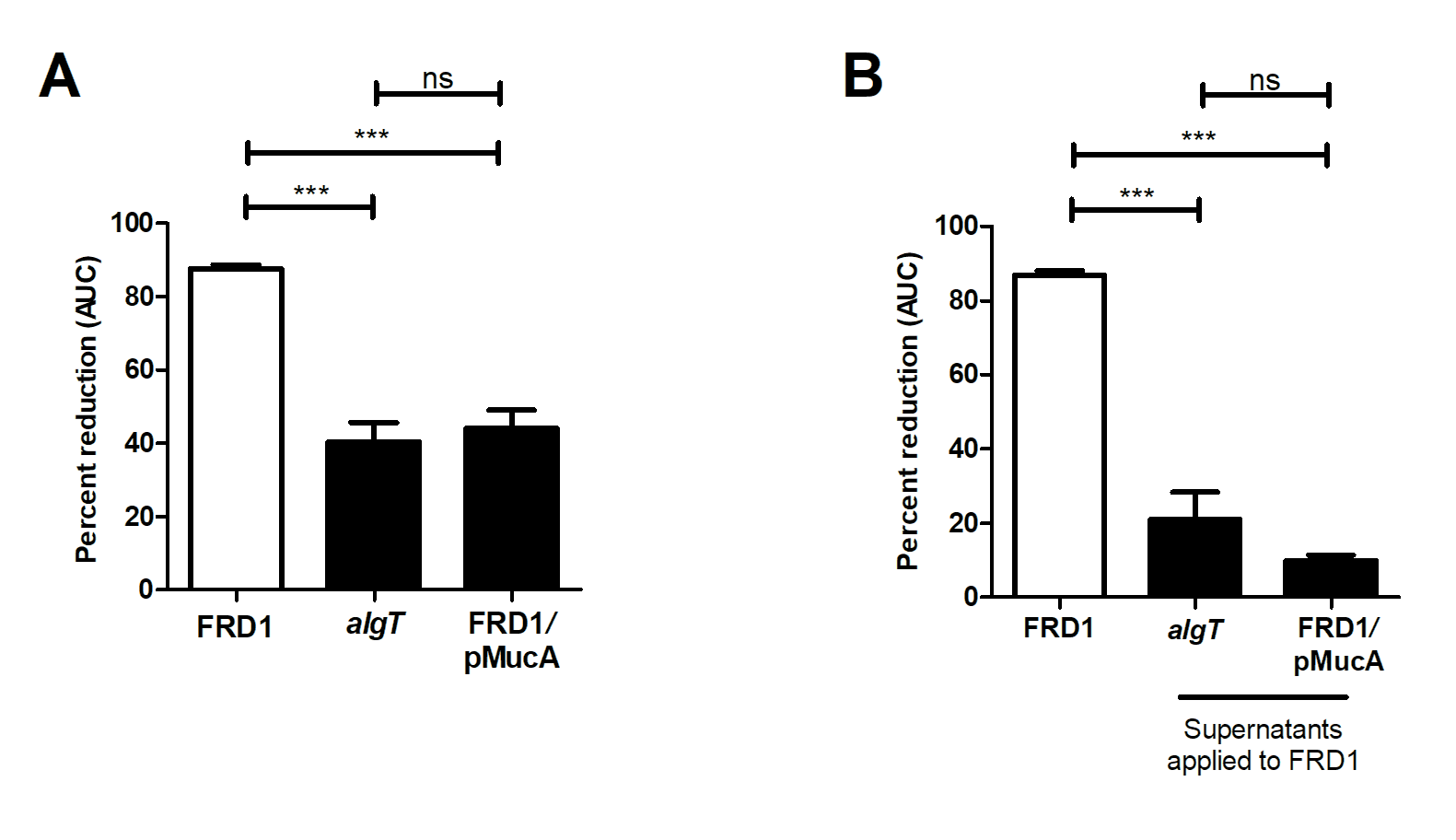

Supplement: FIG S6 [file mbo002183774sf6.tif]
