# Supplementary material for: Mixed Communities of Mucoid and Nonmucoid Pseudomonas aeruginosa Exhibit Enhanced Resistance to Host Antimicrobials
Source: mBio. 2018 Mar 27;9(2):e00275-18. doi: 10.1128/mBio.00275-18 (PMC5874919; doi:10.1128/mBio.00275-18)
Supplement: TABLE S1 [file mbo002183774st1.pdf]

Table S1. Bacterial strains, plasmids, and oligonucleotides used in this study*

Strain or plasmid	Phenotype, genotype and/or description	Sources
<u>*P. aeruginosa* strains</u>		
FRD1	Alg+, FRD1 Δ*mucA22,* clinical isolate	(1)
FRD1*algT*	Alg-, FRD1 Δ*mucA22, algT* D18G, spontaneous nonmucoid revertant of FRD1; also known as FRD2	(1)
FRD1*algD*	Alg-, FRD1 Δ*mucA22, algD::*Tc^R; also known as FRD870	(2)
FRD1-$Strep^R$	FRD1 spontaneous streptomycin resistant isolate	This study
FRD1*algT*-Rif^R	FRD1*algT* spontaneous rifampicin resistant isolate	This study
FRD1*algD*-Rif^R	FRD1*algD* spontaneous rifampicin resistant isolate	This study
Clinical mucoid isolates	Alg+, strains isolated from CF patient sputum samples; Include: CF32, CF43, 2902, 2904, 2919, 2920, 2957, 2959, and 2963	This study
Paired mucoid/*algT* revertant isolates	Strains obtained from O. Ciofu at Univ. of Copenhagen; Include: 6057, 6057*algT*, 6059, 6059*algT*, 6061, 6061*algT*, 6067, 6067*algT*, 6069, 6069*algT*, 6071, 6071*algT*	(3)
algT Δ*katA*	FRD1*algT* with an in-frame, unmarked deletion of *katA*	This study
algT Δ*katB*	FRD1*algT* with an in-frame, unmarked deletion of *katB*	This study
algT Δ*katA/*pHERD	FRD1*algT* Δ*katA* with empty vector pHERD20T	This study
algT Δ*katA/*pKatA	FRD1*algT* Δ*katA* with *katA* in pHERD20T	This study
FRD1*algB*	Alg-, FRD1 Δ*mucA22, algB*::Tn*501*; also known as FRD444	(4)
FRD1*algR*	Alg-, FRD1 Δ*mucA22*, *algR*::Ω sm^R; also known as FRD810	(4)
FRD1*amrZ*	Alg-, FRD1 Δ*mucA22*, *amrZ*::*xylE-aacC1*; also known as FRD1200	(5)
algT Δ*lys*/pHERD	FRD1*algT* Δ*lys* with empty vector pHERD20T	This study
algT Δ*lys*/pLys	FRD1*algT* Δ*lys* with *lys* in pHERD20T	This study
FRD1/pHERD	FRD1 with empty vector pHERD20T	This study
*algT/*pHERD	FRD1*algT* with empty vector pHERD20T	This study
FRD1/pMucA	Alg-, FRD1 with *mucA* in pHERD20T	(6)
<u>*E. coli* strains</u>		
HB101	*recA13 leuB6 ara-14 proA2 lacY1 galK2 xyl-5 mtl-1 rpsL20(SmR) glnV44 λ-*	Laboratory strain
NEB5α	*fhuA2 Δ(argF-lacZ)U169 phoA glnV44 Φ80 Δ(lacZ)M15 gyrA96 recA1 relA1 endA1 thi-1 hsdR17*	New England Biolabs
S17/λpir	*thi recA thr leu tonA lacY supE* RP4–2-Tc::Mu1::*pir* Km^R	Laboratory strain
<u>Plasmids</u>		
pEX18Ap	Ap^R ($Carb^R$); suicide replacement vector containing *B. subtilis sacB* gene	(7)
pSM1	Ap^R ($Carb^R$); pEXAp bearing *P. aeruginosa* in-frame deletion of *katA*	This study
pSM2	Ap^R ($Carb^R$); pEXAp bearing *P. aeruginosa* in-frame deletion of *katB*	This study
pSM3	Ap^R ($Carb^R$); pEXAp bearing *P. aeruginosa* in-frame deletion of *lys*	This study
pHERD20T	pUCP20T P*lac* replaced with 1.3-kb AflII-EcoRI fragment of *araC*-PBAD cassette (5,087 bp)	(8)
pHERD20T-*katA*	*katA* in pHERD20T XbaI/HindIII	This study

pHERD20T-*lys*	*lys* in pHERD20T XbaI/HindIII	This study
pHERD20T-*mucA*	*mucA* in pHERD20T EcoRI/HindIII	(6)
<u>Oligonucleotides</u>		
*katA*del-1	CGGGATCCGTTGAAGAGCAGGAAGACGAG	This study
*katA*del-2	TCATCAGGCCATCAGTCCAG CATTTACTCTCTCCTCAACGGC	This study
*katA*del-3	GCCGTTGAG GAGAGAGTAAATGCTGGACTGAT GGCCTGATGA	This study
*katA*del-4	CCCCAAGCTTGCAAGACCCTGTACATCCTC	This study
*katB*del-1	CGGGATCCATGAAGATGGCGAAGGCCAC	This study
*katB*del-2	GGTTGCGATCAATCCTGGAG CATGGAAGAGCTCCTAATGGC	This study
*katB*del-3	GCCATTAG GAGCTCTTCCATGCTCCAGGATT GATCGCAACC	This study
*katB*del-4	CCCAAGCTTGCCGTTGTCGTGAATGAATC	This study
*katA*comp-1	GCTCTAGATAGCACGTTAGCCGTTGAGG	This study
*katA*comp-2	CCCAAGCTTCAGGCCATCAGTCCAGCTT	This study
*lys*del-1	CGGGATCCTGAATGACCGCCTGCTCAAG	This study
*lys*del-2	CTCATGACAGCACCGCCCT CTGCTCGGTCAGTTTCATCG	This study
*lys*del-3	CGATGAAACTGACCGAGCAG AGGGCGGTGCTGTCATGAG	This study
*lys*del-4	CCCAAGCTTGCATGCTGGCTGTAACCTCT	This study
*lys*comp-1	GCTCTAGA CGCCTGAAACCCATCGGAGT	This study
*lys*comp-2	CCCAAGCTTCTCATGACAGCACCGCCCT	This study
rpoD RT-F2	GCCGAGCTGTTCATGCCGAT	(9)
rpoD RT-R2	GAACAGGCGCAGGAAGTCGG	(9)
algD RT 67-F	TGTGGATGTCTCCAGCACCAAGAT	(9)
algD RT 68-R	AGATGAACGATACGTCGGAGTCCA	(9)
katA RT 10-F	CATCGAGAACCTGACCAACG	This study
katA RT 10-R	TCAGGTCGAACGGGTTGTAG	This study
lys RT 1-F	GAACCTCAATTACAGCGCCC	This study
lys RT 1-R	GTAGGTGTTGTCGGCAATCG	This study

*Alg-, non-mucoid phenotype, Alg+, mucoid phenotype

References

1. Ohman DE, Chakrabarty AM. 1981. Genetic mapping of chromosomal determinants for the production of the exopolysaccharide alginate in a *Pseudomonas aeruginosa* cystic fibrosis isolate. Infect Immun 33:142–148.
2. Ma L, Wang S, Wang D, Parsek MR, Wozniak DJ. 2012. The roles of biofilm matrix polysaccharide Psl in mucoid *Pseudomonas aeruginosa* biofilms. FEMS Immunol Med Microbiol.
3. Ciofu O, Lee B, Johannesson M, Hermansen NO, Meyer P, Høiby N. 2008. Investigation of the *algT* operon sequence in mucoid and non-mucoid *Pseudomonas aeruginosa* isolates from 115 Scandinavian patients with cystic fibrosis and in 88 in vitro non-mucoid revertants. Microbiology 154:103–113.
4. Wozniak DJ, Ohman DE. 1994. Transcriptional analysis of the *Pseudomonas aeruginosa* genes *algR, algB, and algD* reveals a hierarchy of alginate gene expression which is modulated by *algT*. J Bacteriol 176:6007–6014.
5. Wozniak DJ, Sprinkle AB, Baynham PJ. 2003. Control of Pseudomonas aeruginosa *algZ* expression by the alternative sigma factor AlgT. J Bacteriol 185:7297–7300.
6. Limoli DH, Rockel AB, Host KM, Jha A, Kopp BT, Hollis T, Wozniak DJ. 2014. Cationic Antimicrobial Peptides Promote Microbial Mutagenesis and Pathoadaptation in Chronic Infections. PLoS Pathog 10.
7. Hoang TT, Karkhoff-Schweizer RR, Kutchma AJ, Schweizer HP. 1998. A broad-host-range F1p-FRT recombination system for site-specific excision of chromosomally-located DNA sequences: Application for isolation of unmarked *Pseudomonas aeruginosa* mutants. Gene 212:77–86.
8. Qiu D, Damron FH, Mima T, Schweizer HP, Yu HD. 2008. PBAD-based shuttle vectors for functional analysis of toxic and highly regulated genes in *Pseudomonas* and *Burkholderia spp.* and other bacteria. Appl Environ Microbiol 74:7422–7426.
9. Jones CJ, Ryder CR, Mann EE, Wozniak DJ. 2013. AmrZ modulates *Pseudomonas aeruginosa* biofilm architecture by directly repressing transcription of the psl operon. J Bacteriol 195:1637–1644.
