# Supplementary material for: Mixed Communities of Mucoid and Nonmucoid Pseudomonas aeruginosa Exhibit Enhanced Resistance to Host Antimicrobials
Source: mBio. 2018 Mar 27;9(2):e00275-18. doi: 10.1128/mBio.00275-18 (PMC5874919; doi:10.1128/mBio.00275-18)
Supplement: TEXT S1 [file mbo002183774s1.pdf]

Supplemental Materials and Methods

Alginate purification and quantitation. Clinical mucoid isolate, FRD1, was grown on PIA at 37º C for 16-18 hours. Colonies were scraped directly from the solid medium and homogenized in 0.85% saline. After vortexing the bacterial suspension, cells were pelleted by centrifugation at 21,952xg for 30 minutes at room temperature. Alginate was precipitated from the supernatant with 2% cetyl pyridinium chloride and centrifuged at 21,952xg for 10min at room temperature. The pellet was dissolved in cold 100% isopropanol and centrifuged at 21,952xg for 10min at room temperature. The alginate pellet was rehydrated in saline and incubated 12-24h at 4°C.

Purified alginate preparations were quantitated as follows: Brown algae-derived (seaweed) alginate (Sigma) was dissolved in saline and diluted two-fold (2mg/mL to 62.5mg/mL) to generate standards. Alginate standards or sample were placed in duplicate into a 96-well plate. 25 mM sodium tetraborate in sulfuric acid was added to each well, followed by heating on a standard heatblock at 100ºC for 15 minutes. 0.1% carbazole was then added into each well, followed by heating at 100ºC for 15 minutes. After cooling the plate to room temperature, absorbance was read on a Spectramax plate reader at wavelength of 550nm.

Quantitative reverse transcriptase-PCR. Bacterial strains were grown to exponential phase (OD_{600}= ~1.0). Bacterial cells were pelleted by centrifugation, and resuspended/lysed in TRIzol for 5 minutes. Chloroform was added and the aqueous phase was collected and applied to the genomic DNA eliminator column provided in the Qiagen RNeasy Kit. The column was centrifuged at 8000 x g for 30 seconds at 4ºC. RNA was purified following manufacturer's instructions. RNA was eluted with 50uL RNase-free water.

The RNA preparations were converted to cDNA using the Thermo SuperScript III First-Strand Synthesis System, per manufacturer's instructions. Quantitative real time PCR was

performed using Bio-Rad CFX1000 thermal cycler, gene-specific primer pairs, and SYBR green supermix (Biorad). All samples were normalized to housekeeping gene *rpoD* and mRNA transcript fold-change was calculated relative to FRD1.

Catalase activity assays. Supernatants from bacteria were diluted 1:4 in assay buffer and added to a 96 well plate to a final volume of 78µL. The negative control was LBNS. A H_2O_2 standard curve was generated (0, 2, 4, 6, 8, and 10 nmol) to a final volume of 78µL. 10µL of stop solution was added into the wells containing the standards. One additional well included catalase enzyme with stop solution (this was treated as the "high control"). 12µL fresh 1mM H_2O_2 was added into each well to start the reaction and incubated at room temperature for 30m. 10µL of stop solution was then added to each well. A 50µL developer containing 46µL assay buffer, 2µL OxiRed Probe, and 2µL HRP solution was prepared and added to samples. After 10m at room temperature, absorbance was measured at OD_{570nm} using a Spectramax plate reader. Catalase activity was calculated by determining signal change: ΔA= absorbance of the high control-absorbance of each sample. After plotting the standard curve, linear regression was used to convert ΔA to nmol of H_2O_2 decomposed by catalase in a 30m reaction (=B). Catalase activity could then be calculated as follows:

$$Catalase\ activity = \frac{B}{t} \times V \times \ dilution\ factor$$

where t = 30min, V = sample volume (mL), and one unit of catalase decomposes 1µmol of H_2O_2 per minute at pH 4.5 at 25°C.
